# Role of cross-reactivity in cellular immune targeting of influenza A M1_58-66_ variant peptide epitopes

**DOI:** 10.3389/fimmu.2022.956103

**Published:** 2022-09-23

**Authors:** Galina V. Petrova, Yuri N. Naumov, Elena N. Naumova, Jack Gorski

**Affiliations:** ^1^ The Blood Research Institute, Versiti Wisconsin, Milwaukee, WI, United States; ^2^ Department of Pediatrics, Medical College of Wisconsin, Milwaukee, WI, United States; ^3^ Smart Diagnostics Medica, Boston, MA, United States; ^4^ Division of Nutrition Epidemiology and Data Science, Friedman School of Nutrition Science and Policy, Tufts University, Boston, MA, United States

**Keywords:** human, T cells, T cell receptor, pathogen recognition, cross-reactivity

## Abstract

The immunologic significance of cross-reactivity of TCR recognition of peptide:MHC complexes is still poorly understood. We have described TCR cross-reactivity in a system involving polyclonal CD8 T cell recognition of the well characterized influenza viral M1_58-66_ epitope. While M1_58-66_ is generally conserved between influenza A isolates, error-prone transcription generates stable variant RNA during infection which could act as novel epitopes. If packaged and viable, variant genomic RNA generates an influenza quasispecies. The stable RNA variants would generate a new transmissible epitope that can select a specific repertoire, which itself should have cross-reactive properties. We tested two candidate peptides in which Thr65 is changed to Ala (A65) or Ser (S65) using recall responses to identify responding T cell clonotypes. Both peptides generated large polyclonal T cell repertoires of their own with repertoire characteristics and cross-reactivity patterns like that observed for the M1_58-66_ repertoire. Both substitutions could be present in viral genomes or mRNA at sufficient frequency during an infection to drive immunity. Peptides from the resulting protein would be a target for CD8 cells irrespective of virus viability or transmissibility. These data support the hypothesis that cross-reactivity is important for immunity against RNA virus infections.

## Introduction

T cell receptor (TCR) cross-reactivity is a concept that describes the interaction of a unique TCR with different peptide-MHC complexes. While it has predominantly been studied as a laboratory phenomenon, it could have practical consequences. TCR cross-reactivity is part of a multiple mapping phenomenon characterizing the antigen presentation and recognition process. Antigen presentation and recognition involves three highly variable molecules, polymorphic MHC, distinct antigen peptides, and the highly variable yet clonotypic TCR. These three molecules are involved in four mapping scenarios: 1) a unique MHC allele can bind (map to) multiple peptides; 2) a single peptide can be bound by multiple MHC alleles; 3) multiple peptide-MHC complexes can be recognized by a single TCR; and 4) a single peptide-MHC complexes can be recognized by multiple TCR. Important inroads into the biophysical understanding of TCR cross-reactivity have been made (1–6), although much yet needs to be clarified.

Recognition of multiple peptides with conservative replacements in the context of the same MHC describes the simplest TCR cross-reactive system in which one TCR can recognize many peptides in one MHC. This mapping generates a potential for recognition of viral escape variants. These variants will predominantly utilize conservative substitutions so as not to interfere with the function of the protein. TCR cross-reactivity has an immunological correlate in thymic maturation. The low-affinity recognition of self during positive selection leaves space for multiple potential peptide structures, so that pathogen-derived epitopes can be recognized in the future. Viewed in this manner, pathogen-derived peptides are escape variants of self, and because of cross-reactivity, the panoply of pathogen-derived peptides recognized by a single TCR is extensive. Thus, the question whether simple cross-reactivity involving different peptides within the same MHC can be selected for as part of an immune response is of general interest.

In this communication we focus on the immune-response aspect of TCR cross-reactivity. The cross-reactivity involves recognition of the same MHC, to which one of a series of similar peptides is bound (defined as mapping scenario 3 above). The T cells come from subjects who are known to have developed T cell memory to one (or more) of these peptides. We define T cell memory as the capability of responding to antigenic peptides in recall cultures. Because many different TCR clonotypes are involved, we are also examining the mapping of different TCR to the same peptide-MHC (pMHC), defined as mapping scenario 4 above.

Specifically, we examine cross-reactivity involving different peptides in the context of immunity to influenza A virus, a common respiratory pathogen causing seasonal epidemics and occasional pandemics with high mortality and morbidity (7–13). Immune response to influenza consists of both humoral (antibodies) and cytotoxic (cellular) responses. The antibody response is generally against surface proteins in the viral coat and is leveraged in most influenza vaccines. Humoral immunity involves CD4 T cell help for the initial B cell selection as well as any further tuning of antibody responses by somatic hypermutation (14). Cytotoxic T cell responses to influenza are generally directed against epitopes from virus-derived internal proteins and presented by MHC class I molecules. In HLA-A2 positive individuals, who make up a large portion of the US population (15), cytotoxic CD8 T cell response to influenza is preferentially directed against the matrix-derived M1_58-66_ epitope (16, 17) which is highly conserved in influenza A strains (18, 19). The CD8 T cells responding to M1_58-66_ predominantly utilize the BV19 TCR, whose CDR3 length is 11 (L11) and whose sequence encodes a canonical “RS” amino acid motif at CDR3 positions 5 and 6 (20–22). Analysis of CDR3 sequences of the CD8 BV19 T cell repertoire responding to this epitope indicated that this repertoire is polyclonal (23, 24). Further studies demonstrated the self-similar power law-like structure in M1_58-66_ repertoire. A power law-like distribution is scalable, as the number of data points increases (or decreases) the distribution’s shape remains the same. We have proposed that a power law-like distribution arises from replicative expansion (2 to the power of n) of a normally distributed collection of receptors (naïve TCR) for a fixed ligand (pMHC), with high-affinity receptors expanding more frequently (25). We had also shown that power law-like distributions are indicative of a fractal system and associated with the flexibility and robustness of the immune response (26, 27).

We have shown that the polyclonal M1_58-66_ repertoire includes a high frequency of cross-reactive clonotypes (28, 29). Up to 50% of M1_58-66_-specific clonotypes can proliferate and expand after stimulation with M1 peptides substituted at TCR contact positions. The structural basis of TCR cross-reactivity in this system has been investigated (5, 30, 31). Interestingly, our analysis of M1 cross-reactivity showed that the extent of cross-reactivity, as defined by the number of additional peptides recognized vs. fraction of clonotypes in that category also fits a power law-like distribution (28). This indicates that if M1 is the selected target many clonotypes only recognize it, and as the number of additional peptides recognized by a clonotype increases, the number of clonotypes involved drops off precipitously.

Why the immune system generates such a polyclonal and cross-reactive repertoire in response to this relatively conserved epitope is still poorly understood. One possibility involves robustness of the immune response after thymic involution when the pool of naïve T cells is low. A cross-reactive system would optimize adult recognition of novel epitopes (32). A novel epitope can come from a virus crossing into humans from an unknown reservoir. Alternatively, it could result from escape by an established pathogen. Influenza A is an example of the latter. Variants may arise due to RNA polymerase errors during synthesis of genomes and/or mRNA. Variation can be compounded by the action of RNA editing enzymes. The resultant amino acid substitutions could be immunogenic independent of their effect on viral fitness simply because MHCI restricted peptides are derived from newly synthesized proteins regardless of their fitness (33) and presented as long as the modified peptides do not affect binding to the MHCI. Stable genome RNA variants can lead to a virus population that is a distribution of closely related species, called a quasispecies (34, 35), which could also be a target for CD8 recognition. In any of these cases, actual exposure to a variant during or after the initial exposure should generate a memory T cell response to the variant. Such a memory repertoire should have all the characteristics of the repertoire to M1_58-66_ including cross-reactivity. An overview of the paper is provided as **Supplemental Figure S1**.

In this study, we asked whether the recall response data can provide insight into the possible selection of cross-reactive T cells in response to non-infective variants or quasispecies. We present the analysis of adult TCR repertoire characteristics, including cross-reactivity, after *in vitro* stimulation of PBMC with two candidate M1 peptides substituted at TCR contact residues. These substituted peptides generate polyclonal repertoires with similar characteristics to those associated with the accepted M1_58-66_ epitope. The key is whether the repertoires for A65 and S65 are skewed in favor of self-recognition and if the extent of cross-reactivity shows a power law-like distribution, with many clonotypes only recognizing the target and the number of clonotypes recognizing more than one peptide dropping off precipitously. As might be expected of real-world conditions, there are interesting differences in details of the cross-reactivity observed. The discussion includes a recent report on the identification of the A65 variants, A65, in the influenza isolate (36).

## Materials and methods

### Study subjects

Three healthy middle-aged HLA-A2.1 blood donors (Donors A, B, and C, aged 37, 38 and 47 years old, respectively) were enrolled under protocols authorized by the Institutional Review Board of BloodCenter of Wisconsin (Now Versiti): BC 04-22, “Robust T Cell Immunity to Influenza in Human Populations.”

### Peptides

Influenza A matrix M1_58-66_ peptide (M1) and M1-substituted peptides were synthesized by standard solid-phase methods, purified by HPLC, and confirmed by mass spectrometry (Peptide Core Lab, Blood Research Institute, Versiti Wisconsin). We generated single amino acids substitutions in the M1_58-66_ peptide (GILGFVFTL) at TCR contact positions 63 (Val) and 65 (Thr). Thr_65_ was substituted for Gly, Ala, and Ser.Val_63_ was substituted for Thr, Leu, and Ile. Peptides were named based on the amino acid substitution and position of substitution. For example: if Thr_65_ was substituted for Ser (S), then the substituted peptide is S65 (GILGFVFSL). The A65 and S65 peptides are considered potential epitope candidates.

### Cytotoxic T cell Lines (CTL) generation, RNA isolation, and cDNA preparation

PBMC from buffy coats of three healthy middle-aged HLA-A2.1 donors were isolated, cryopreserved, and stored as previously described (28). CTLs were established in triplicate for each peptide according to described protocol (28) and cultured for 2 weeks. CD8 T cells were isolated using Dynal^®^ CD8 Positive Isolation Kit (Invitrogen, Carlsbad, CA). RNA isolation was performed using Dynabeads^®^ mRNA DIRECT^™^ Kit (Invitrogen). cDNA was synthesized immediately after RNA isolation using oligo(dT) as primer and M-MLV (Invitrogen) as reverse transcriptase. The BV19 gene was amplified from cDNA using BV19-specific primer and fluorochrome-labeled TCR CB primer as described (37). For Donor C, CTLs for peptides substituted at position 63 were cultured for 3 weeks without following CD8 isolation. For comparison, M1_58-66_ and negative control cultures (no peptide) were performed in the same manner. As described previously (28) only PCR products with evidenced of focused CDR3 lengths were further examined.

### PCR product cloning, sequencing, and data analysis

PCR product was subcloned using TOPO TA Cloning^®^ Kit for Sequencing (Invitrogen, Carlsbad, CA), and 96–144 colonies from each sample were randomly chosen and prepared for sequencing as previously described (28). Sequencing was performed by either AGENCourt Bioscience Corp. (Beckman Coulter Company, Beverly, MA) or GeneWiz, Inc. (South Plainfield, NJ). Data were analyzed using FinchTV software (Geospiza, Inc., Seattle, WA). Clonotypes were named on the basis of their amino acid sequence with a numerical coding that allows reconstruction of the nucleotide sequence (38). All the clonotypes used in this study along with the level of response to the various peptides are included in **Supplemental Table S1**.

### Repertoire characteristics

The data utilized here consist of TCR sequences which express the BV19 TCR and of CDR3 length of 11, or of length 10 with J1.2 use. These are characteristics of a major subset of T cells involved in the response. A unique CDR3 nucleotide sequence defines a clonotype, which is the result of a gene rearrangement process. The number of bacterial colonies with the same sequence generates a count of the clonotype. The clonotype data used for this paper are available as **Supplemental Table S1**. General repertoire measurements and characteristics were defined as previously described (27, 39). For each donor and each of three repertoires (M1, A65 and S65), the following measurements were used in this study:

M – number of sequences analyzed,

N – number of unique clonotypes identified,

Ns – number of clonotypes that appeared only once (singletons),

N_RS_ – number of clonotypes with RS CDR3 motif,

N_RSs_ – number of singletons with RS CDR3 motif,

M_RS_ – number of copies of clonotypes with RS CDR3 motif,

N_CR_ – number of cross-reactive clonotypes.

N_NON-CR_ - number of non-cross-reactive clonotypes

Based on these measurements we derived the following characteristics:

N_RS_/N – fraction of clonotypes with RS CDR3 motif,

N_RSs_/Ns – fraction of singletons with RS CDR3 motif among total singletons,

M_RS_/M – proportion of RS clonotypes,

N_CR_/N – fraction of cross-reactive clonotypes.

We ranked all clonotypes in a repertoire based on the number of copies for each clonotype, plotted, and calculated their relative frequency in each rank as a fraction of the corresponding peptide- and donor-specific repertoire. Parameters for the repertoire power law-like distribution were estimated as described previously (26). The contribution of the cross-reactive clonotypes was calculated as N_CR_/N.

### Repertoire analysis

We transformed the rank and rank-frequency values using natural logarithms, normalized in a range from 0 to 1, selected critical points for each repertoire and calculated slope and intercept values by regressing log-frequencies against log-ranks in the first component of the fractal distribution as described previously (26, 40). To estimate the coefficient and standard error for slope and intercept we applied a mixed effects model as described elsewhere (41). For each parameter, the lower and upper confidence intervals were estimated as: coefficient ± standard error.

### Cross-reactivity analysis

We used a rank-repertoire-number summary to estimate the cross-reactivity (28). We ranked all clonotypes based on the number of peptide repertoires in which they were identified. Thus, clonotypes that were found in only one peptide repertoire were ranked as “1”; clonotypes found in the repertoire in question and in any one other peptide repertoire were ranked as “2”; and so on. The relative frequency of clonotypes at each rank was regressed against the fraction of the peptide and donor-specific repertoire. A simple cross-reactivity metric, connecting the clonotypes recognizing only two peptides is plotted as a simple graph for each donor and all donors.

## Results

The results are divided into three sections. The first deals with the clonotype distribution and characteristics of the A65 and S65 repertoires, as compared to the M1 repertoire (Sections 3.1 – 3.3). The second characterizes the cross-reactivity of these repertoires (3.4 – 3.6). The final characterizes the private, non-cross-reactive, portion of each repertoire (3.7).

### Description of the M1, A65 and S65 repertoires

**Figure 1:**
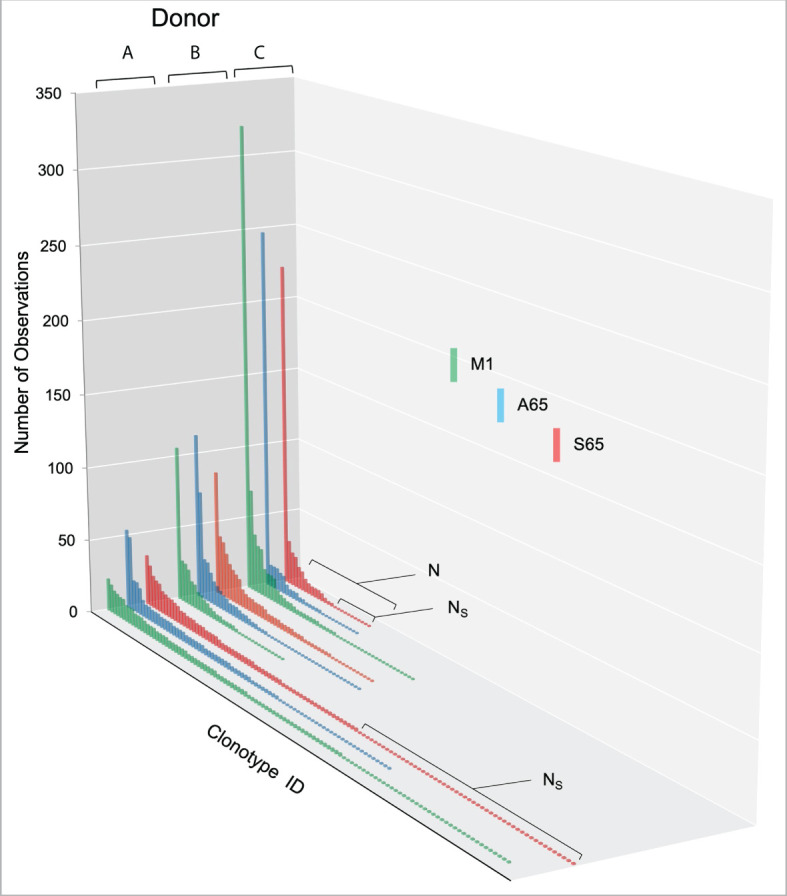
Distribution of clonotypes in M1, A65 and S65 peptide repertoires for three donors. Donors A, B and C are identified above each clonotype distribution, and peptides are identified by color as shown in the legend. Clonotype frequencies are plotted in descending order and shown on the y-axis. Clonotype IDs for each donor are on the z-axis and omitted for simplicity. N – the number of clonotypes in a repertoire; Ns – the number of singletons. The repertoire characteristic values are provided in **Table 1**.

We are using *in vitro* expansion as our measure of response and quantifying the number of specific TCRβ cDNA as the expansion measure. HLA-A2-M1_58-66_ specific T cells predominantly utilize BV19 and have restricted CDR3 length and amino acid sequence restriction. We have shown previously that M1-specific T cells that are cross-reactive on A65 and S65 have similar BV19-CDR3 properties. Here we characterize the A65 and S65 repertoires. For each peptide, the clonotypes expanded in triplicate two-week cultures were ranked by number of observations for the three donors examined and are shown in **Figure 1**. M1 data is also included for comparison. For each donor, the BV19 repertoires responding to M1, A65 or S65 generated clonotype repertoires similar to each other in the repertoire shape. The major differences between donors occur in the extent of expansion of some of the clonotypes (left portion of data set in **Figure 1**). The expansion difference is in part a function of exposure history. Summaries of the repertoire measurements for all three peptides tested are provided in **Table 1**.

**Table 1:** Repertoire characteristics and summaries for M1_58-66_ and substituted peptides for three donors.

Donor	Donor A			Donor B			Donor C		
Peptide	M1	A65	S65	M1	A65	S65	M1	A65	S65
Number of clonotypes identified, N	110	77	115	33	49	47	49	28	26
Number of sequences analyzed, M (sum of number of observations)	398	395	451	305	404	424	653	366	381
Number of clonotypes that appeared only once (singletons), Ns	40	30	52	14	27	12	22	11	11
Number of clonotypes with RS CDR3 motif, N_RS_	71	64	79	20	37	27	32	21	19
Number of singletons with RS CDR3 motif, N_RSs_	24	25	35	9	17	5	15	7	7
Number of non-cross-reactive clonotypes identified, N_NON-CR_	53	27	55	14	32	24	31	14	9
Number of cross-reactive clonotypes identified, N_CR_	57	50	60	19	17	23	18	14	17
Fraction of clonotypes that appeared only once (singletons), Ns/N	0.36	0.39	0.45	0.42	0.55	0.26	0.45	0.39	0.42
Fraction of clonotypes with RS CDR3 motif, N_RS_/N	0.65	0.83	0.69	0.61	0.76	0.57	0.65	0.75	0.73
Fraction of non-cross-reactive clonotypes, N_NON-CR_/N	0.48	0.35	0.48	0.42	0.65	0.51	0.63	0.50	0.35
Fraction of cross-reactive clonotypes, N_CR_/N	0.52	0.65	0.52	0.58	0.35	0.49	0.37	0.50	0.65
Average fraction of clonotypes with RS CDR3 motifs (N_RS_/N)	0.72 ± 0.09			0.65 ± 0.10			0.71 ± 0.05		
Average fraction of non-cross-reactive clonotypes, N_NON-CR/N_	0.44 ± 0.07			0.53 ± 0.12			0.49 ± 0.14		
Average fraction of cross-reactive clonotypes, N_CR_/N	0.56 ± 0.07			0.47 ± 0.12			0.51 ± 0.14		

### Rank – rank frequency analysis

A graph of the clonotype ID versus the count of that clonotype shows a long tail of clonotypes counted once or a few times. Deeper insight is gained by counting the frequency of unique counts. We refer to this unique clonotype count as the rank property of the clonotype. The rank serves as a proxy for previous expansion and thus for abundance. The rank frequency is a proxy for richness of the repertoire. The plot of rank vs rank frequency generates a clonotype distribution that approximates the diversity of the repertoire. Rank can be a predictor of capability of expansion in culture since high-ranking clonotypes became high ranking by expanding *in situ*. We have described previously that in adults the recall repertoire rank-frequency distributions often appear to have two components, a power law-like component at lower ranks and a second component of high ranking clonotypes, with one clonotype per rank. We have previously shown by directly sequencing T cells from PBMC that the *ex vivo* BV19 CD8 repertoire has a similar two-component distribution. Furthermore, by analyzing samples drawn at multiple times, we observed that the second component is always present and represents a temporally stable portion of the circulating repertoire (40). This was also true for the L11 “RS” encoding portion of the *ex vivo* BV19 repertoire.

**Figure 2:**
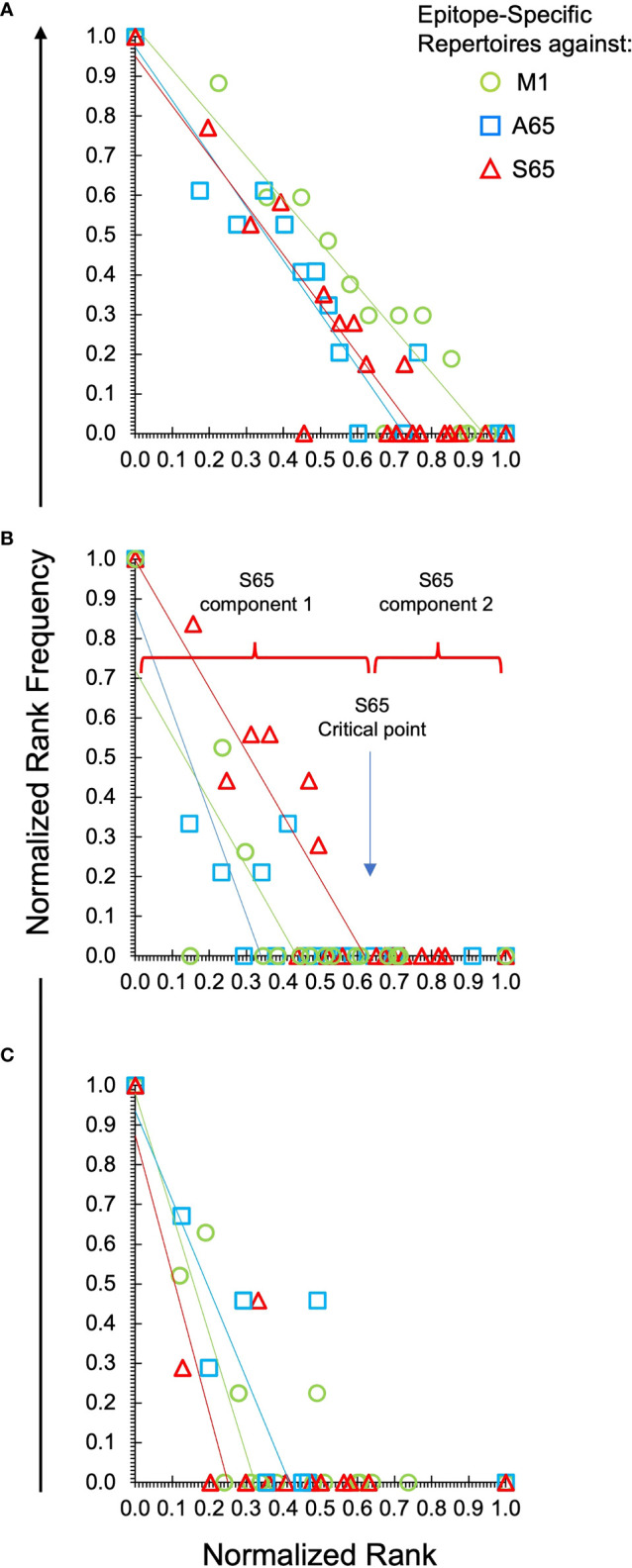
Power law-like distribution of clonotypes in each repertoire of each donor and peptide. Natural log-transformed values are fit to a linear-regression line for the first component of the repertoire. Panel **(A)** is data from Donor A. Panel **(B)** for Donor B and Panel **(C)** for Donor C. Stimulating peptides are identified by marker color as shown in the legend in panel **(A)**. The division of the data into the power law-like component 1 and component 2 are shown for the S65 data of Donor B. Details are provided in **Supplemental Table S2**.

To facilitate comparisons of the different peptides, we normalize the natural log-log transformed data and show the repertoire summaries for all three peptides on the same graph (**Figure 2**). The two-component distribution is most obvious in Donor B and C as the data represent a “broken-stick.” An example of the two components and the critical point constituting the boundary between the two is shown for the S65 in panel B. The donor C plots seldom have a first component beyond 0.4 normalized rank. Donor A data all break above 0.75 normalized rank and are thus predominantly characterized by the power law-like (first) component. The repertoire analysis, which includes the regression analysis of the slopes and the intercepts for the power law-like component as well as the critical point, are provided in **Supplemental Table S2**. The average of the pooled regression models, including average coefficients for the slope and intercept parameters indicating the overall similarity of the three peptide repertoires for each donor are provided in **Table 2**. The overlapping values of both slope and intercept parameters across donors for all three peptides indicate a high degree of similarity. Thus, the clonotype distributions of the three repertoires are similar for each donor and for the combined peptide repertoires from all three donors. If a power law-like distribution is indicative of robustness, then complex repertoires can tolerate some level of changes in pathogenic peptide, as far as these changes do not alter binding to MHC and do not create significant changes in peptide-MHC structure.

**Table 2:** Regression models of *epitope-specific* repertoires: the average values of slopes and intercepts across three peptides for each donor are shown.

Donor	Parameters	Coefficients	*Standard* error	*t*-statistic	*p*-value	Lower confidence interval	Upper confidence interval
A	intercept	0.911	0.073	12.572	0.000	0.754	1.069
	slope	-1.032	0.113	-9.241	0.000	-1.276	-0.789
B	intercept	0.579	0.107	5.616	0.001	0.351	0.807
	slope	-0.832	0.192	-4.569	0.003	-1.242	-0.423
C	intercept	0.531	0.136	3.908	0.005	0.225	0.837
	slope	-0.797	0.284	-2.857	0.023	-1.436	-0.158

Details for each peptide are provided in **Supplemental Table 2**.

### Utilization of RS clonotypes and other repertoire characteristics

**Figure 3:**
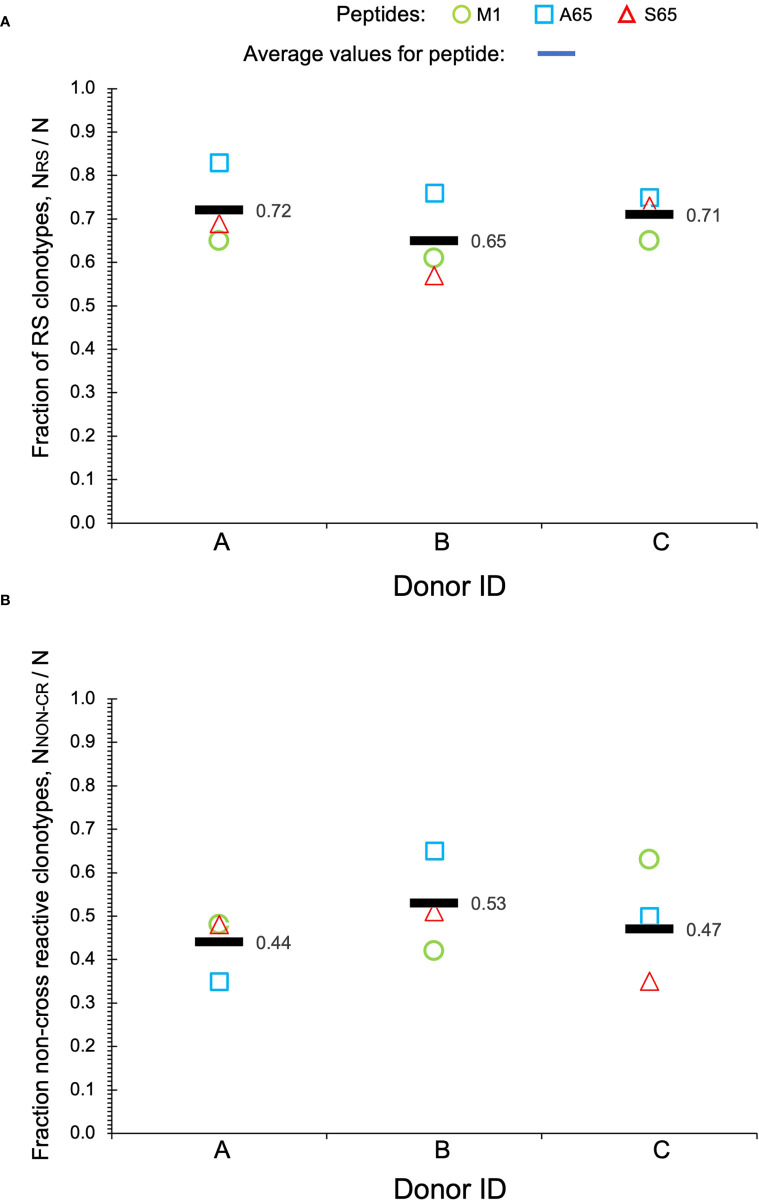
Repertoire characteristics. **(A)** Fraction of RS clonotypes calculated as N_RS_/N in the BV19 repertoires responding to the two query peptides and M1 in the three donors. Averages are shown by bars. **(B)** Fraction of clonotypes in the non-cross-reactive component to the repertoires. Data is available in **Table 1**.

Clonotypes with the RS amino acid motif are frequently observed in response to M1 and are considered to be recruited during the period of thymic activity (39). We would expect that TCR recognizing substitutions in the M1 epitope would continue to use the RS CDR3 motif. We enumerated the total RS clonotypes, N_RS_ and calculated the fraction of RS clonotypes in the total BV19 repertoire, N_RS_/N, for each peptide. The average RS usage for M1 and the two other putative epitopes were 0.72, 0.65 and 0.71 for the three donors respectively (**Figure 3A**). The high RS utilization characteristic of the three peptides, with one being a recognized epitope, makes it likely that the other two may also be “epitopes.” We use the term epitope in the immunological sense, that it is a part of a pathogen-derived protein that is recognized by the adaptive immune system and is the foundation for future recognition (memory).

Other characteristics of the three repertoires such as the number of singletons are given in **Table 1**. Of interest is the extent to which the three repertoires are private, i.e. non-cross-reactive. As part of these studies, we stimulated the PBMC from the three donors with four additional M1 peptides, G63, L63, I 63 and T63, which represent conservative substitutions. The fraction of non-cross-reactive peptides (N_NON-CR/_N) for each of the three repertoires with respect to the other six peptides is shown in **Figure 3B**. For each donor, the A65 and S65 non-cross-reactive repertoire values are similar to that of M1. The non-cross-reactive repertoires will be evaluated in more detail later, and the cross-reactive components analyzed next.

### Cross-reactivity of A65 and S65 repertoires

Cross-reactivity is a characteristic of a clonotype. A repertoire is cross-reactive to the extent that it is composed of cross-reactive clonotypes. Thus, a cross-reactive clonotype belongs to more than one repertoire. Experimentally, a clonotype is considered cross-reactive if it participates in the recall response to more than one peptide. Using a panel of six peptides substituted at position 63 or 65, we have shown that the contribution of M1_58-66_ cross-reactive clonotypes was ~ 50% (28). Using the same panel of peptides, we examined the cross-reactivity of the A65 and S65 repertoires. Each repertoire is defined by all the clonotypes that responded to that peptide. The data used is provided in **Supplemental Table S1**. The clonotype data for the M1, A65 and S65 repertoires is complete. However, for G65, L63, I63 and T63 only the cross-reactive component of the repertoire is shown. The fraction of the A65 cross-reactive clonotypes, as defined by clonotypes that recognize A65 and another of the G65, L63, I63, and T63 peptides, varied from 0.35 for Donor B to 0.50 and 0.65 for Donors C and A, respectively. The contribution of the S65 cross-reactive clonotypes varied from 0.49 for Donor B to 0.53 and 0.63 for Donor A and C. These data are summarized in **Table 1** labelled N_CR_/N.

**Figure 4:**
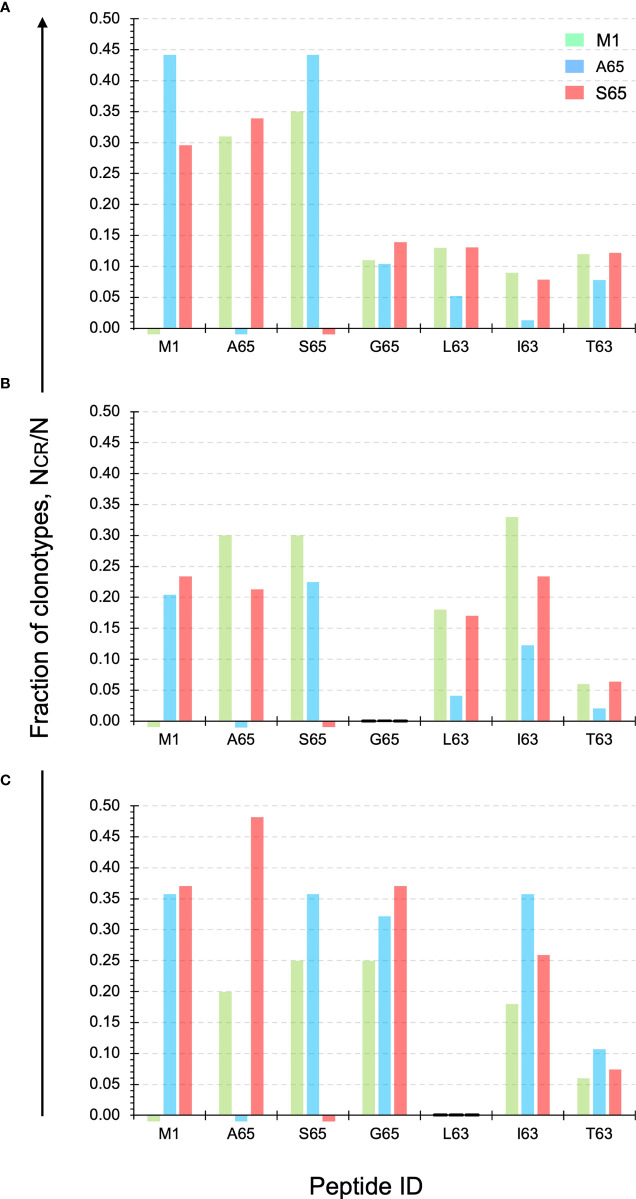
Cross-reactivity of the M1, A65, and S65 repertoires. Each panel ID, **(A–C)** corresponds to the donor ID. The y-axis shows the fraction of clonotypes from the two candidate and M1 repertoires that are also present in the repertoires identified on the x-axis. The M1 and candidate repertoires are identified by color. The relative fraction for the M1 and candidate repertoires compared against themselves, which is “1”, is omitted for clarity. Data is provided in **Supplemental Table S3**.

The exact structure of the cross-reactivity for the M1 repertoire as well as the two epitope candidates (A65 and S65) for each donor is shown in **Figure 4**. The y-axis shows the fraction of clonotypes from the two candidate and M1 repertoires that are also present in the repertoires identified on the x-axis. The M1 and candidate repertoires are identified by color. Thus, the red bar over the I63 position on the x-axis, shows the fraction of the S65 repertoire (red) that was also in the I63 repertoire. The relative fraction for the M1 and candidate repertoires compared against themselves, which is “1”, is omitted for clarity. A small stub below the x-axis shows the position of the M1, A65, and/or S65 repertoires for which no response to that peptide was detected.

For Donor A the level of cross-reactivity for the four additional peptides is similar for the M1 and S65 repertoires. For L63 and T63 the clonotype fraction is identical. Cross-reactivity of the A65 repertoire shows a greater of variation, with I63 showing the lowest level of cross-reactivity. The actual fraction for each peptide within the three repertoires and for each Donor is provided in **Supplemental Table S3**.

For Donor B, none of the three repertoires show cross-reactivity with the G65 peptide. For the remaining three peptides, the S65 and M1 repertoires show similar or equal levels of cross-reactivity for the L63 and T63 peptides. For I63 the levels for M1- and S64-associated clonotypes are more variable.

For Donor C, it is now the L63 peptide that is not recognized in either of the three repertoires. G65 is highly represented in all three of the repertoires. I63 is also recognized to a great extent by the candidate epitopes. T63 is the least recognized by clonotypes from the three repertoires. These results once again show the similarity of the repertoires generated by the two epitope candidates in terms of the recognized epitope. The pattern of reactivity observed in **Figure 4** suggested that an analysis of the number of peptides recognized and the connectivity of the cross-reactive clonotypes should be informative.

### Quantifying the extent of cross-reactivity of the A65 and S65 repertoires

**Figure 5:**
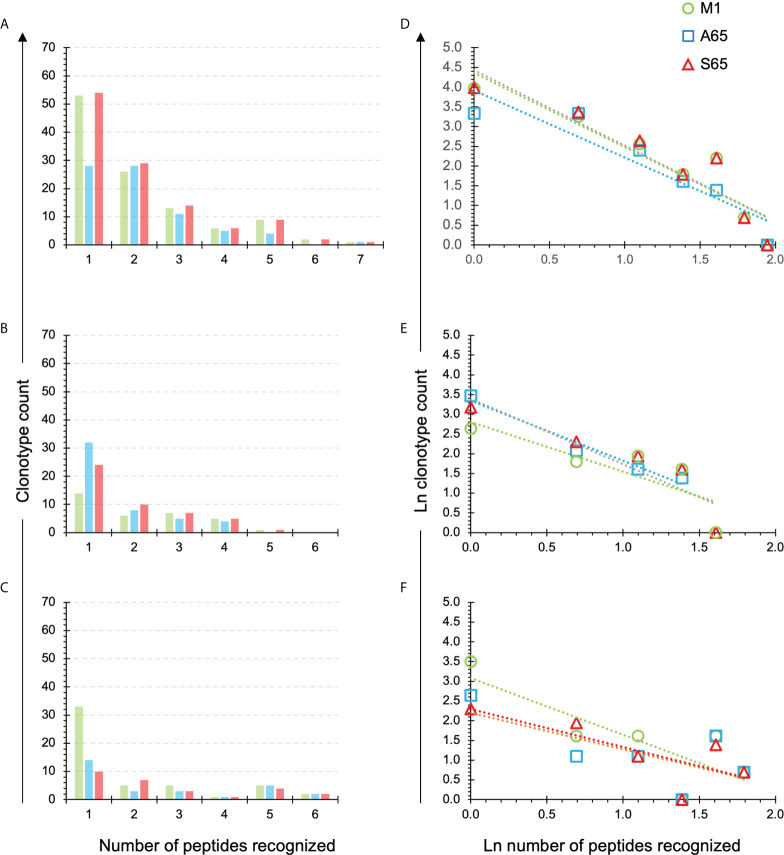
Extent of cross-reactivity of clonotypes in the M1, A65, and S65 repertoires. The repertoires are identified by color. **(A–C)** correspond to the eponymous donors and show the counts of the M1, A65 or S65 reactive clonotypes that recognize one or more peptides. The number of peptides recognized is shown on the x-axis. An x value of one represents the non-cross-reactive repertoire. A value of seven represents a clonotype that was present in all repertoires. **(D–F)** show ln rank vs. ln rank-frequency plots of the data in **(A–C)** respectively. Details of the power law-like component are provided in **Supplemental Tables S4** and **S5** aggregated by donor or peptide respectively. Average values from the Supplemental Tables are in **Tables 3**, **4** respectively.

We have previously introduced the quantification of cross-reactivity in terms of the number of peptide repertoires in which a clonotype is identified (28). A clonotype recognizing only one peptide, or observed in one repertoire, is not cross-reactive. A clonotype recognizing all seven peptides would be fully cross-reactive for this dataset. The number of clonotypes in the M1, A65, S65 repertoires recognizing one to seven peptides is shown for each donor in **Figures 5A–C**, respectively. For Donor A, the maximum number of peptides recognized by a single clonotype was seven, and this for all three repertoires. For Donor B and Donor C, the maximum was six.

We plotted the natural log of the count of clonotypes against the natural log of the number of peptides recognizing to determine if the cross-reactivity of A65 and S65 clonotypes follows a power law-like distribution (**Figures 5D–F**). The fraction of recognized clonotypes is at its maximum when only one peptide is recognized then decreases as the number of peptides increases. This was the case for all three donors (**Figures 5D–F**). These results are similar for all three peptides in a donor-specific manner.

To demonstrate the similarity of the clonotype distributional profiles with respect to the cross-reactivity for clonotypes responding to M1, A65 and S65, we estimated the slope or intercept using a mixed effects model in which the values obtained from each donor were treated as an individual cluster. The confidence intervals for the slope or intercept parameters allow their comparison across the variants. A summary of the average slope and intercept of the three repertoires is shown in **Table 3**. The estimated parameters for each of the peptide-based distributions were overlapping indicating that the distributional profiles of all three donors are similar. A detailed description for each peptide and each donor is provided in **Supplemental Table S4**. The *p*-value for Donor C A65 is greater than 0.05. The *R^2^* values for A65 and S65 for Donor C indicate a poor fit. However, in light of the limited dataset, a power law-like distribution provides a reasonable approximation for the extent of cross-reactivity.

**Table 3:** Results of regression models of *cross-reactive* repertoires: the average values of slopes and intercepts for each donor are shown.

Donor	Parameters	Coefficients	Standard error	*t*-statistic	*p*-value	Lower confidence interval	Upper confidence interval
A	intercept	3.754	0.449	8.267	0.003	2.516	4.993
	slope	-1.474	0.281	-5.013	0.012	-2.230	-0.719
B	intercept	2.667	0.343	9.786	0.009	1.505	3.828
	slope	-1.093	0.279	-4.624	0.046	-2.054	-0.132
C	intercept	2.528	0.589	4.289	0.014	0.891	4.164
	slope	-1.111	0.471	-2.351	0.086	-2.417	0.196

Details for each peptide are provided in **Supplemental Table 3**.

To ensure that each donor exhibited internal consistency in the distributional profiles across the variant peptides, we repeated the analysis to compare the peptide-specific average slopes and intercepts. We expected that internal consistency will be reflected by the standard error of the coefficients: the smaller the error, the higher the consistency. The peptide averages of the slope and intercept are shown in **Table 4**. Details of the peptide-based aggregation is provided in **Supplemental Table S5**. Based on the overlapping confidence intervals for the slope and intercept values we conclude that peptide-specific profiles are similar.

**Table 4:** Results of regression models of *cross-reactive* repertoires: the average values of slopes and intercepts for each peptide.

Peptide	Parameters	Coefficients	Standard error	*t*-statistic	*p*-value	Lower confidence interval	Upper confidence interval
M1	intercept	3.416	0.556	6.403	0.008	1.827	5.006
	slope	-1.529	0.452	-3.630	0.046	-2.829	-0.228
A65	intercept	2.676	0.404	9.116	0.012	1.404	3.949
	slope	-1.027	0.283	-4.491	0.059	-1.934	-0.119
S65	intercept	2.856	0.421	6.824	0.006	1.681	4.031
	slope	-1.123	0.296	-3.867	0.038	-1.938	-0.307

Details for each peptide-donor combination are provided in **Supplemental Table 4**.

Thus, the analysis of the cross-reactivity for the clonotype repertoires generated by the A65 and S65 peptides demonstrated that cross-reactivity can be approximated by a power law-like distribution and that data for a slope and an intercept are similar for all three peptides in a donor-specific manner and for all three donors in a peptide-specific manner.

### Connectivity of M1, A65 and S65 repertoires

**Figure 6:**
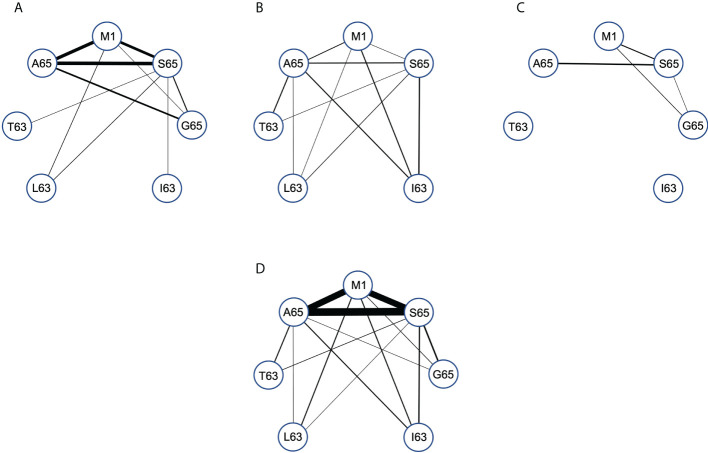
Pair-wise comparison of repertoire connectivity for the M1, A65, and S65 repertoires. The thickness of each connection also reflects this number. **(A–C)** Represent the connectivity for Donors **(A–C)** respectively. **(D)** Is the combined connectivity for all repertoires. Connectivity values are given in **Supplemental Table S6**.

We compared the paired cross-reactive connectivity pattern for the peptides M1, A65, S65 and G65 (**Figure 6**) and included any cross-reactivity of these the position 65 repertoires with peptides that varied at position 63. In our previous analysis of the M1 repertoire (28), we included all clonotypes in the pair-wise connectivity. However, the data in **Figure 5** show that there are some clonotypes that show extensive cross-reactivity. These will bias a pairwise examination. For example, there are two clonotypes in the Donor C repertoires that have an extent of cross-reactivity of six and thus each would contribute five connections on a pairwise graph. To eliminate such bias **Figure 6** is restricted to only those clonotypes with an extent of cross-reactivity equal to two. This generates a simple undirected graph (two peptides connected by one clonotype). The thickness of the connector reflects the number of clonotypes making the connection. The connectivity data is available in **Supplemental Table S6**. Panels A, B and C in **Figure 6** represent the data for each of the donors and Panel D is the connectivity of the entire data set.

Examining the graph for the entire data set summary (**Figure 6D**) shows an incomplete and unbalanced connectivity pattern. M1 and A65 have at least one connection to the other six peptides. A65 lacks a connection to I63. The most extensive connectivity (largest number of connections) is between M1 and A65 and S65 (M1-A65 = 14, M1-S65 = 12, A65-S65 = 19). All three have a much-reduced connectivity to G65. Thus, the number of cross-reactive clonotypes examined in this pair-wise manner is very similar for M1_58-66_, A65 and S65 peptide repertoires but different for G65. If G65 were an independent epitope, we would expect to see more G65 clonotypes that cross-react with either M1_58-66_ or A65 or S65. There is a reduced level of cross-reactivity with the peptides substituted at position 63. For A65, S65 and G65, these peptides have two differences in sequence, the position 63 difference and the position 65 difference. Despite this increased divergence, there is still cross-reactivity evidenced between A65 and S65 and the position 63 substituted peptides.

The summary analysis reveals the possible extent of connectivity in naturally derived clonotypes, when the population is three subjects. However, connectivity has a donor-specific nature (**Figures 6A–C**). Donor A has the strongest three-way pattern between M1_58-66_, and A65 and S65. There is recognition of the position 63 substituted peptides, but predominantly from the S65 repertoire, which connects to all four. Donor B has a weaker connection between A65 and M1, but a relatively balanced connectivity pattern. Each of the three repertoires has at least two connections to the other four peptides, with the S65 repertoire again connecting to all four. Donor C has the simplest connectivity pattern, which has a strong connection between M1 and T63, and a few others. Donor specificity in the cross-reactive patterns generated in the recall repertoire, could be a function of thymic T cell selection in the face of other HLA differences, including the differences in naïve repertoire. However, this could also reflect the different exposure histories of the donors. Exposure history could include differences in disease progression, innate response, mutant generation, as well as strain differences. This topic is taken up in the discussion.

### The non-cross-reactive portions of the M1_58-66_, A65 and S65 repertoires

**Figure 7:**
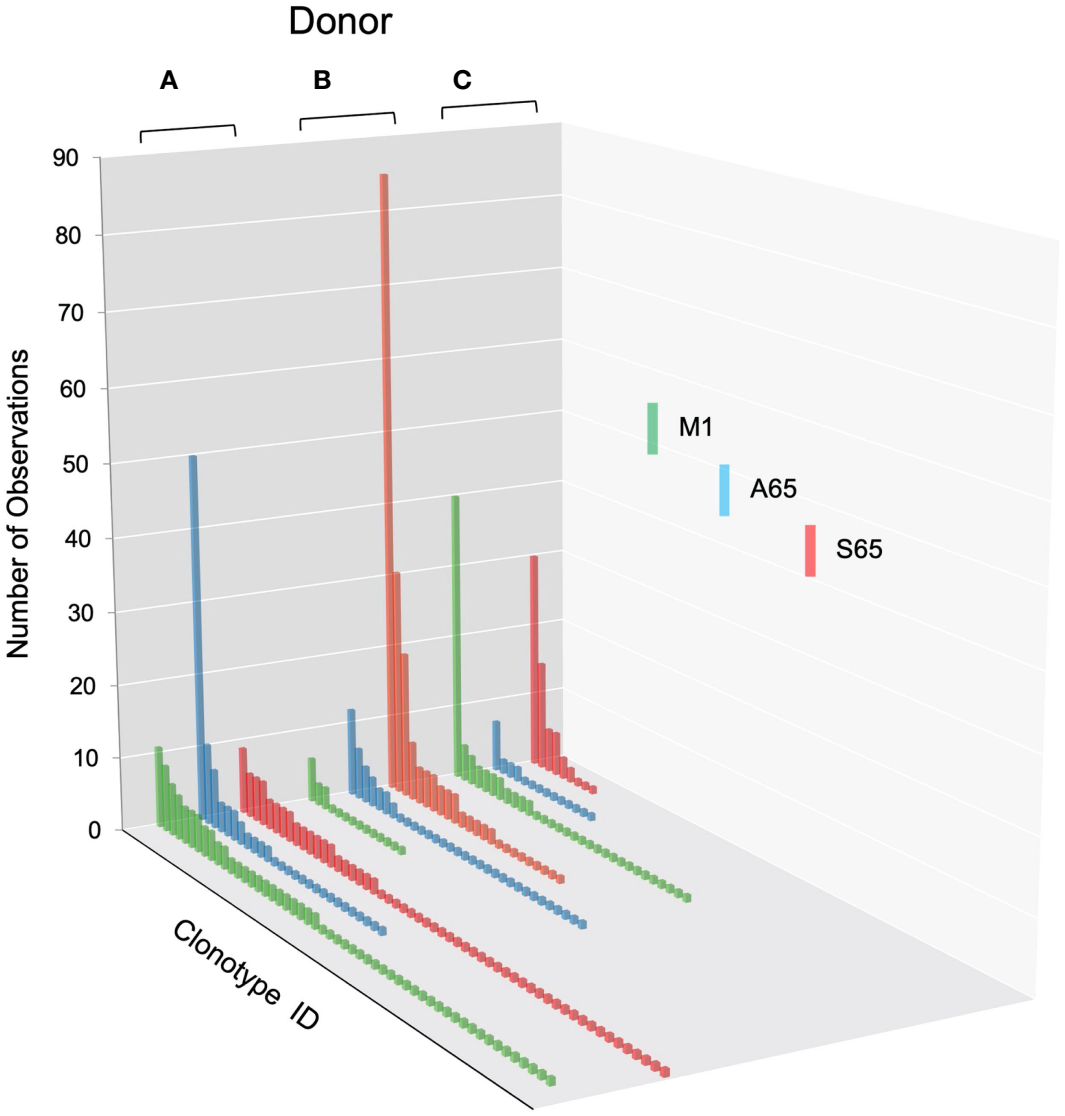
Clonotype distributions for non-cross-reactive clonotypes from M1, A65, and S65 peptide repertoires for all three donors. Donors A, B and C are identified above each clonotype distribution, and peptides are identified by color as shown in the legend. Clonotype frequencies are plotted in descending order and shown on the y-axis. Clonotype IDs for each donor are on the z-axis and omitted for simplicity. The repertoire characteristic values are provided in **Table 5**.

The clonotypes making up the non-cross-reactive portions of the A65 and S65 repertoires are similar to that of M1 and contain high-frequency clonotypes. In the previous section, we showed how the cross-reactive components of the M1, A65 and S65 repertoires are similar. We would expect this portion of the repertoire to be similar as well. The non-cross-reactive clonotype distributions of the three repertoires for the three donors are shown in **Figure 7**.

The distributions for non-cross-reactive repertoires differ in the extent and frequency of high-ranking clonotypes. We expect cross-reactivity to be a mass action phenomenon. A mass action mechanism assumes that a fraction of clonotypes can be cross-reactive with another related peptide in the same MHC. This should be easily observed if there is a high frequency of observations of the primary response. Thus, an expanded clonotype that recognizes M1 and has a ten percent chance of recognizing A65 will appear in the A65 repertoire if it has expanded sufficiently to allow the 10% to be measured. In keeping with this phenomenon, most high frequency clonotypes are cross-reactive. This is also true of the repertoires of both the other epitope candidates. However, there are some counterexamples for each donor. The third highest frequency clonotype in A65 repertoire of Donor A repertoire is non-cross-reactive. For Donor B, the second and third highest clonotypes in the M1 repertoire are non-cross-reactive. For Donor C the third highest frequency clonotype in the M1 and S65 repertoires is non-cross-reactive. The most likely explanation is that we did not offer the correct cross-reactive peptide for that clonotype. It is also possible that these observations may point to a non-mass action mechanism. Nevertheless, observing high frequency clonotypes in the A65 or S65 repertoires private repertoires (not cross-reactive) argues for the possibility that the peptide is an epitope.

The number of RS clonotypes and the number of observations of RS clonotypes is similar in the non-cross-reactive component of the M1, A65, and S65 repertoires (**Table 5**). This is also the case when the data are expressed as the fraction of RS clonotypes and the proportion of all observations. This can be compared to the same repertoire characteristics for the non-cross-reactive repertoires generated by the three position 63 peptides. For these three peptides, the fraction of RS clonotypes is three- to ten-fold lower than in the A65 or S65 repertoires. The difference in the proportion of observations due to RS clonotypes is 10- to 40-fold lower in the position 63 repertoires as compared with the M1 repertoire. Thus, the non-cross-reactive portions of the A65 and S65 repertoires are similar to the corresponding total repertoires and to the M1 repertoire.

**Table 5:** *Non-cross-*reactive repertoire characteristics and summaries for each donor and each peptide.

Donor	Donor A			Donor B			Donor C		
Peptide	M1	A65	S65	M1	A65	S65	M1	A65	S65
Number of clonotypes identified, N	53	27	55	14	32	24	31	14	9
Number of sequences analyzed, M (sum of number of observations)	117	108	108	23	61	190	89	23	65
Number of clonotypes that appeared only once (singletons), Ns	29	16	35	11	25	9	20	10	3
Number of clonotypes with RS CDR3 motif, N_RS_	33	20	37	7	22	12	18	11	6
Number of singletons with RS CDR3 motif, N_RSs_	17	12	23	7	15	3	13	7	1
Fraction of clonotypes that appeared only once (singletons), Ns/N	0.55	0.59	0.64	0.79	0.78	0.38	0.65	0.71	0.33
Fraction of clonotypes with RS CDR3 motif, N_RS_/N	0.62	0.74	0.67	0.50	0.69	0.50	0.58	0.79	0.67
Average fraction of clonotypes that appeared only once (singletons), Ns/N	0.59 ± 0.04			0.65 ± 0.24			0.71 ± 0.20		
Average fraction of clonotypes with RS CDR3 motifs (N_RS_/N)	0.72 ± 0.09			0.65 ± 0.10			0.71 ± 0.05		

## Discussion

Adaptive T cell responses are valued for their specificity yet the underlying biophysics of the TCR:pMHC interaction allows for cross-reactivity. This is no small feat. We have argued that a robust immune system would include cross-reactivity as a buffer against epitope variants (28, 32). We measured such cross-reactivity for the recall response to the well-characterized influenza A M1_58-66_ epitope, taking advantage of the highly polyclonal nature of this response. However, influenza is an RNA virus with all the characteristics pertaining thereto; and it is likely that the exposure history of our subjects reflects this pathogen complexity. Therefore, we examined the possibility that some of the more theoretical aspects of our cross-reactivity experiments may have uncovered actual variant M1_58-66_ epitopes that drive T cell selection and increase the level of cross-reactivity.

We tested two candidate peptides, A65 and S65, in the context of whether these peptides are epitopes. Since the peptide with T65 is the accepted epitope, we compared these two candidates to the wild-type M1_58-66_. The two candidate peptides are very similar to M1_58-66_ in the three parameters and measures that we have examined (**Figures 1**–**3**). The cross-reactivity of these two repertoires is as broad as that of M1_58-66_. Most importantly, the A65 and S65 repertoires have extensive non-cross-reactive clonotypes whose characteristics are similar to that of the non-cross-reactive M1_58-66_ repertoire in terms of polyclonality, clonotype distribution, and CDR3 sequence composition. These data are compatible with the possibility that these variations are naturally occurring, and that the immune system has generated a strong recall repertoire against them in the donors analyzed.

An important caveat to this work is that the data were generated prior to introduction of single-cell analysis and hence rely on the β-chain only as a definition of clonotype. The β-chain lineage is defined early during thymic ontogeny in a stage referred to as β-chain selection. After this stage, during which the β-chain is paired with an α-chain surrogate, there is an expansion of the selected cell prior to the beginning of α−chain rearrangement. Hence the β−chains examined here that are associated with different peptide responses could be in part associated with different α-chains (42). Resolving this issue is an important next stage in this form of recall analysis, as would be an accompanying transcriptome analysis. The existing single-cell data for the M1 response (5) is not large enough to determine the relation between β- and α-chain pairing. However, we examined two larger single-cell data sets (~2K cells), one from colon-resident CD8 cells (43), and the other splenic CD4 cells specific for an LCMV peptide (44). The percent of clonotypes that were accounted for by cells sharing a β-chain and showing exclusive α-chain pairing was 2.3% for the CD8 and 2.4% for the CD4 repertoires (**Supplemental Figure S2**). Thus, we can assume that <3% of the cross-reactive data in our dataset may be attributable to responses by independent α-β clonotypes.

We propose that the A65 and S65 M1 variants arise during an infection and add to the available epitope pool, independent of the viability of the viral progeny that would use the resultant viral RNA (45). They may become part of the quasispecies generated by the infection and can be present in the initial exposure of another individual. In that case they would be an initial epitope. Indeed, the A65 substitution has been shown to be viable by Rimmelzwaan and colleagues (46). The variant viral RNAs are packaged as particles (47) during the height of infection and the immune system can focus on them to the same extent. Some of these variants may become established as actual substrains and occur at frequencies sufficient for their sequence to be measurable in analyses of viral isolates. In this regard, a search of the current influenza databases revealed a report of the A65 variant in a patient sample (36) [NCBI Sequence Source: AZU90740.1]. For such an observation to be made as part of routine analysis this should be the major sequence component of the virus isolate. Future use of high-resolution genome sequencing of influenza virus populations and/or infected cells should resolve the details about the nature of the influenza populations in an infection.

The quantitative analyses of diversity (rank-rank frequency plots) and cross-reactivity are donor independent for A65, S65 and M1_58-66_ and thus can be considered a general phenomenon. However, there is an important element of donor specificity in the qualitative analyses in clonotype characteristics and of the connectivity of the cross-reactive responses. Donor-specific variability can be expected in a recall analysis of middle-aged individuals as polymorphic differences in cellular and immune functions could lead to differences in the extent of viral variation.

Exposure history may be of equal, if not greater, importance for explaining donor-specific repertoires. Exposure history is unlikely to be identical in terms of virus strains propagating at the time of exposure. An example from our data is the increased I63 cross-reactivity in Donors A and B, which could be due to exposure to a viral mixture in which this variant was present. A search of protein sequence databases (48–50) identified possible reservoirs in more than 50 bird and swine influenza A strains and one human isolate with an M1 sequence corresponding to I63 [NCBI Sequence Source: AHL99571.1]. An encounter by with such a virus by Donors B and C, but not Donor A may explain the reactivity pattern in **Figure 4**. It is also a likely explanation for the increased use of RS clonotypes in the I63 repertoire of Donors B and the high frequency use of RS in all the repertoires from Donor C.

A general pattern of reactivity such as seen for A65 and S65 might support a cellular variant model, in which these variations arise in any cell infected by a wild-type M1 strain. The more specific patterns might argue for a presence of the variant in the infecting virus population. However, irrespective of which variant scenario or combination of scenarios might be the case, our data strongly suggest that the CD8 arm of the immune system can focus on variant peptides. We have recently generated evidence in a sentinel study that in HLA-A2 individuals the CD8 response can be important in helping resolve or prevent morbidity due to natural influenza exposures (in preparation) and thus the ability of CD8 T cell repertoires to encompass variant virus could be an important facet of such protection. It would also represent the continued selection for a “fuzzy” biophysical recognition system in the T cell arm of the adaptive response.

In addition to the immediate involvement with evolution of dynamic pathogens, cross-reactivity could be needed for the temporal evolution of a responding repertoire (i.e. aging). We have already shown that with time the RS clonotypes involved in M1 responses become replaced with other clonotypes (39). This is a likely result of expansion-contraction cycles associated with recurring exposures (25, 27, 51). We propose that the “new” flu-responsive clonotypes are a component of another repertoire but have sufficient cross-reactivity to become involved in the response to influenza epitopes. During the decline of an initially selected repertoire with the concomitant increase in inflammation, new clonotypes can re-adjust their signal thresholds (52) and start to expand in this new role.

Finally, allo-reactivity, which is the underlying bane of transplantation, is a very strong form of cross-reactivity focused predominantly on recognition of a different MHC. This is an important medical problem, but the underlying role of cross-reactivity is not often considered. Thus, the biophysics of the cross-reactive phenomenon appears to play a central role in many aspects of host immunity and transplantation.

## Data availability statement

The original contributions presented in the study are included in the article/**Supplementary Materials**. Further inquiries can be directed to the corresponding author.

## Ethics statement

The adult subjects were enrolled under protocols authorized by the Institutional Review Board of BloodCenter of Wisconsin: BC 04-22, “Robust T Cell Immunity to Influenza in Human Populations.”

## Author contributions

GP was involved in recall analyses, in organizing the experiments and collecting and analyzing data. YN was involved in data analysis and writing the paper. EN implemented and performed the high-level analyses and participated in writing the paper. JG was responsible for the overall study design and writing the paper. All authors have read and approved the manuscript.

## Funding

This work was supported by grant U19 AI062627 (JG) from National Institutes of Health and by NRSA training grant 5 T32 HL 7209-35 (GP).

## Acknowledgments

We thank members of the Gorski lab and our other colleagues for discussions of this topic, and the reviewers for helping us clarify our writing.

## Conflict of interest

Author YN was employed by company Smart Diagnostics Medica.

The remaining authors declare that the research was conducted in the absence of any commercial or financial relationships that could be construed as a potential conflict of interest.

## Publisher’s note

All claims expressed in this article are solely those of the authors and do not necessarily represent those of their affiliated organizations, or those of the publisher, the editors and the reviewers. Any product that may be evaluated in this article, or claim that may be made by its manufacturer, is not guaranteed or endorsed by the publisher.

## Supplementary material

Supplementary Table 1 Clonotype data used in the paper. Data for each donor is shown on a separate sheet. Starting at Row 18, the columns provide the: Donor ID, clonotype name, four amino acid motif starting at CDR3 position 4, nucleotide sequence, extent of cross reactivity (X-CR), canonical motif ID, and counts for the clonotypes in the respective peptide repertoires identified in each of the next seven columns. The data are sorted by M1 and X-CR, then by A65 and X-CR, and then by S65 and X-CR. Only M1, A65 and S65 include the entire repertoire. Data in the remaining columns shows the count of that clonotype in the respective G65, L63, I63, T63 repertoire only if the clonotype was already present in the M1, A65 and/or S65 repertoire(s). The headers provide summary values for the data in the columns equivalent to that used in .

Supplementary Figure 1 Overview of paper.

Supplementary Figure 2 Estimating the frequency of clonotypes with identical β-chain rearrangements and unique exclusive α-chain rearrangements. Single-cell T cell analysis from published data (43, 44) was analyzed for the occurrence of clonotypes with the same β-chain gene rearrangement but different α-chain gene rearrangements. Data on the left is from colon-derived CD8 T cells, and that on the right for tetramer selected CD4 T cells participating in a response to LCMV. TCR sequences and cell barcodes were obtained from the deposited files. Clonotypes were identified using a diagonal mapping of cells vs chains. **(A)** Clonotype rank vs. rank frequency distribution shows the two-component structure with an extensive power law-like component. **(B)** An example of two clonotypes with this characteristic from the colon CD4 data set. **(C)** Number of clonotypes in this category (Nβα) and the fraction of the total (Nβα/N). **(D)**. Cell number in each example of such clonotypes. Note that only pairs were identified. No examples of the same β-chain with three mutually exclusive α-chains were observed. The highest cell frequency was three and four cells. Most of these clonotypes were comprised of one or two cells. For comparison, the fractions of clonotypes in which the β-chain could be paired with two different α-chains were 0.146 and 0.140, respectively.
