# Supplementary material for: Role of cross-reactivity in cellular immune targeting of influenza A M158-66 variant peptide epitopes
Source: Front Immunol. 2022 Sep 23;13:956103. doi: 10.3389/fimmu.2022.956103 (PMC9539824; doi:10.3389/fimmu.2022.956103)
Supplement: Supplementary file 2 [file Table_2.docx]

**Supplemental Table S2.** Results of regression models of epitope-specific repertoires: the individual values of slopes and intercepts across three peptides for each donor.

| **Donor** | | **Donor A** | | | **Donor B** | | | **Donor C** | | |
| --- | --- | --- | --- | --- | --- | --- | --- | --- | --- | --- |
| **Peptide** | | **M1** | **A65** | **S65** | **M1** | **A65** | **S65** | **M1** | **A65** | **S65** |
| Intercept | Coefficients | 1.0242 | 0.8456 | 0.8642 | 0.4569 | 0.4560 | 0.8243 | 0.5100 | 0.6493 | 0.4334 |
|  | Standard Error | 0.0674 | 0.0712 | 0.0811 | 0.1230 | 0.1039 | 0.0942 | 0.1158 | 0.1563 | 0.1368 |
|  | *t*-statistic | 15.1919 | 11.8712 | 10.6518 | 3.7133 | 4.3876 | 8.7469 | 4.4036 | 4.1542 | 3.1675 |
|  | *p*-value | <0.0001 | <0.0001 | <0.0001 | 0.0023 | 0.0005 | <0.0001 | 0.0006 | 0.0043 | 0.0090 |
|  | LCI^*^ | 0.8786 | 0.6904 | 0.6930 | 0.1930 | 0.2345 | 0.6245 | 0.2616 | 0.2797 | 0.1322 |
|  | UCI^**^ | 1.1699 | 1.0008 | 1.0354 | 0.7208 | 0.6775 | 1.0241 | 0.7584 | 1.0189 | 0.7345 |
| Slope | Coefficients | -1.0894 | -0.9639 | -1.0441 | -0.7191 | -0.6758 | -1.1021 | -0.8032 | -0.8781 | -0.7096 |
|  | Standard Error | 0.0977 | 0.1212 | 0.1212 | 0.2295 | 0.1879 | 0.1580 | 0.2330 | 0.3377 | 0.2811 |
|  | *t*-statistic | -11.1557 | -7.9525 | -8.6147 | -3.1333 | -3.5971 | -6.9758 | -3.4478 | -2.6001 | -2.5240 |
|  | *p*-value | <0.0001 | <0.0001 | <0.0001 | 0.0073 | 0.0026 | <0.0001 | 0.0039 | 0.0354 | 0.0283 |
|  | LCI | -1.3004 | -1.2279 | -1.2998 | -1.2114 | -1.0762 | -1.4370 | -1.3029 | -1.6767 | -1.3283 |
|  | UCI | -0.8784 | -0.6998 | -0.7884 | -0.2269 | -0.2753 | -0.7672 | -0.3036 | -0.0795 | -0.0908 |
| Multiple *R* | | 0.9515 | 0.9168 | 0.9020 | 0.6420 | 0.6805 | 0.8675 | 0.6776 | 0.7009 | 0.6056 |
| *R^2^* | | 0.9054 | 0.8405 | 0.8136 | 0.4122 | 0.4631 | 0.7526 | 0.4592 | 0.4913 | 0.3667 |
| Observations | | 15 | 14 | 19 | 16 | 17 | 18 | 16 | 9 | 13 |
| Critical Point | | 0.67 | 0.76 | 0.59 | 0.35 | 0.41 | 0.47 | 0.49 | 0.49 | 0.33 |

^*^ - Lower Confidence Interval, ^**^ - Upper Confidence Interval
