# Supplementary material for: Role of cross-reactivity in cellular immune targeting of influenza A M158-66 variant peptide epitopes
Source: Front Immunol. 2022 Sep 23;13:956103. doi: 10.3389/fimmu.2022.956103 (PMC9539824; doi:10.3389/fimmu.2022.956103)
Supplement: Supplementary file 3 [file Table_3.docx]

**Supplemental Table S3**. Cross-reactivity of the M1, A65, and S65 repertoires.

| **Donor** | **Repertoires** | **Fraction of the clonotypes in repertoires responded to peptides** | | | | | | | |
| --- | --- | --- | --- | --- | --- | --- | --- | --- | --- |
|  |  | **M1** | **A65** | **S65** | **G65** | **L63** | **I63** | **T63** | **All X-R** |
| Donor A | M1 | 1 | 0.31 | 0.35 | 0.11 | 0.13 | 0.09 | 0.12 | 0.52 |
|  | A65 | 0.44 | 1 | 0.44 | 0.10 | 0.05 | 0.01 | 0.08 | 0.64 |
|  | S65 | 0.30 | 0.34 | 1 | 0.14 | 0.13 | 0.08 | 0.12 | 0.53 |
| Donor B | M1 | 1 | 0.30 | 0.30 |  | 0.18 | 0.33 | 0.06 | 0.58 |
|  | A65 | 0.20 | 1 | 0.22 |  | 0.04 | 0.12 | 0.02 | 0.35 |
|  | S65 | 0.23 | 0.21 | 1 |  | 0.17 | 0.23 | 0.06 | 0.49 |
| Donor C | M1 | 1 | 0.20 | 0.25 | 0.25 |  | 0.18 | 0.06 | 0.35 |
|  | A65 | 0.36 | 1 | 0.36 | 0.32 |  | 0.36 | 0.11 | 0.50 |
|  | S65 | 0.37 | 0.48 | 1 | 0.37 |  | 0.26 | 0.07 | 0.63 |
