# Supplementary material for: Role of cross-reactivity in cellular immune targeting of influenza A M158-66 variant peptide epitopes
Source: Front Immunol. 2022 Sep 23;13:956103. doi: 10.3389/fimmu.2022.956103 (PMC9539824; doi:10.3389/fimmu.2022.956103)
Supplement: Supplementary file 5 [file Table_5.docx]

**Supplemental Table S5.** Results of regression models of cross-reactive repertoires aggregated to peptides: the individual values of slopes and intercepts across three donors for each peptide.

| **Peptide** | | **M1** | | | **A65** | | | **S65** | | |
| --- | --- | --- | --- | --- | --- | --- | --- | --- | --- | --- |
| **Donor** | | **Donor A** | **Donor B** | **Donor C** | **Donor A** | **Donor B** | **Donor C** | **Donor A** | **Donor B** | **Donor C** |
| Intercept | Coefficients | 4.3580 | 2.8039 | 3.0876 | 2.4793 | 3.3477 | 2.2024 | 4.4253 | 1.8487 | 2.2939 |
|  | Standard Error | 0.4620 | 0.5611 | 0.6462 | 0.4118 | 0.1890 | 0.6099 | 0.4734 | 0.2782 | 0.5121 |
|  | *t*-statistic | 9.4328 | 4.9972 | 4.7780 | 6.0205 | 17.7166 | 3.6108 | 9.3481 | 6.6444 | 4.4793 |
|  | *p*-value | 0.0002 | 0.0154 | 0.0088 | 0.0092 | 0.0032 | 0.0225 | 0.0002 | 0.0069 | 0.0110 |
|  | LCI^*^ | 3.1703 | 1.0182 | 1.2934 | 1.1687 | 2.5347 | 0.5089 | 3.2084 | 0.9632 | 0.8721 |
|  | UCI^**^ | 5.5456 | 4.5895 | 4.8817 | 3.7899 | 4.1607 | 3.8959 | 5.6422 | 2.7341 | 3.7158 |
| Slope | Coefficients | -1.8803 | -1.2602 | -1.4451 | -0.6310 | -1.5260 | -0.9234 | -1.9119 | -0.4931 | -0.9632 |
|  | Standard Error | 0.3365 | 0.5039 | 0.5160 | 0.1616 | 0.1989 | 0.4870 | 0.3448 | 0.1333 | 0.4089 |
|  | *t*-statistic | -5.5882 | -2.5009 | -2.8005 | -3.9049 | -7.6715 | -1.8960 | -5.5454 | -3.6990 | -2.3554 |
|  | *p*-value | 0.0025 | 0.0876 | 0.0488 | 0.0298 | 0.0166 | 0.1308 | 0.0026 | 0.0343 | 0.0781 |
|  | LCI | -2.7453 | -2.8638 | -2.8777 | -1.1453 | -2.3819 | -2.2757 | -2.7982 | -0.9174 | -2.0986 |
|  | UCI | -1.0154 | 0.3434 | -0.0124 | -0.1167 | -0.6701 | 0.4288 | -1.0257 | -0.0689 | 0.1722 |
| Multiple *R* | | 0.9284 | 0.8221 | 0.8138 | 0.9141 | 0.9834 | 0.6880 | 0.9274 | 0.9056 | 0.7623 |
| *R^2^* | | 0.8620 | 0.6758 | 0.6622 | 0.8356 | 0.9671 | 0.4733 | 0.8601 | 0.8202 | 0.5811 |
| Observations | | 7 | 5 | 6 | 5 | 4 | 6 | 7 | 5 | 6 |

^*^ - Lower Confidence Interval, ^**^ - Upper Confidence Interval
