# Supplementary material for: Role of cross-reactivity in cellular immune targeting of influenza A M158-66 variant peptide epitopes
Source: Front Immunol. 2022 Sep 23;13:956103. doi: 10.3389/fimmu.2022.956103 (PMC9539824; doi:10.3389/fimmu.2022.956103)
Supplement: Supplementary file 6 [file Table_6.docx]

**Supplemental Table S6.** Connectivity datasets.

| **Epitope combination** | **Connectivity** | | | |  |
| --- | --- | --- | --- | --- | --- |
|  | **Donor A** (Figure 6A) | **Donor B** (Figure 6B) | **Donor C** (Figure 6C) | **Three Donors** (Figure 6D) |  |
|  |  |  |  |  |  |
| M1-A65 | 14 | 2 | 0 | 16 |  |
| M1-S65 | 10 | 1 | 3 | 14 |  |
| M1-G65 | 0 | 0 | 2 | 2 |  |
| M1-L63 | 2 | 1 | 0 | 3 |  |
| M1-I63 | 0 | 2 | 0 | 2 |  |
| M1-T3 | 0 | 0 | 0 | 0 |  |
| A65-S65 | 13 | 3 | 3 | 19 |  |
| A65-G65 | 1 | 0 | 0 | 1 |  |
| A65-L63 | 0 | 1 | 0 | 1 |  |
| A65-I63 | 0 | 1 | 0 | 1 |  |
| A65-T63 | 0 | 1 | 0 | 1 |  |
| S65-G65 | 3 | 0 | 1 | 4 |  |
| S65-L63 | 2 | 2 | 0 | 4 |  |
| S65-I63 | 0 | 3 | 0 | 3 |  |
| S65-T63 | 1 | 1 | 0 | 2 |  |
