# Supplementary figures and images for: Role of cross-reactivity in cellular immune targeting of influenza A M158-66 variant peptide epitopes
Source: Front Immunol. 2022 Sep 23;13:956103. doi: 10.3389/fimmu.2022.956103 (PMC9539824; doi:10.3389/fimmu.2022.956103)

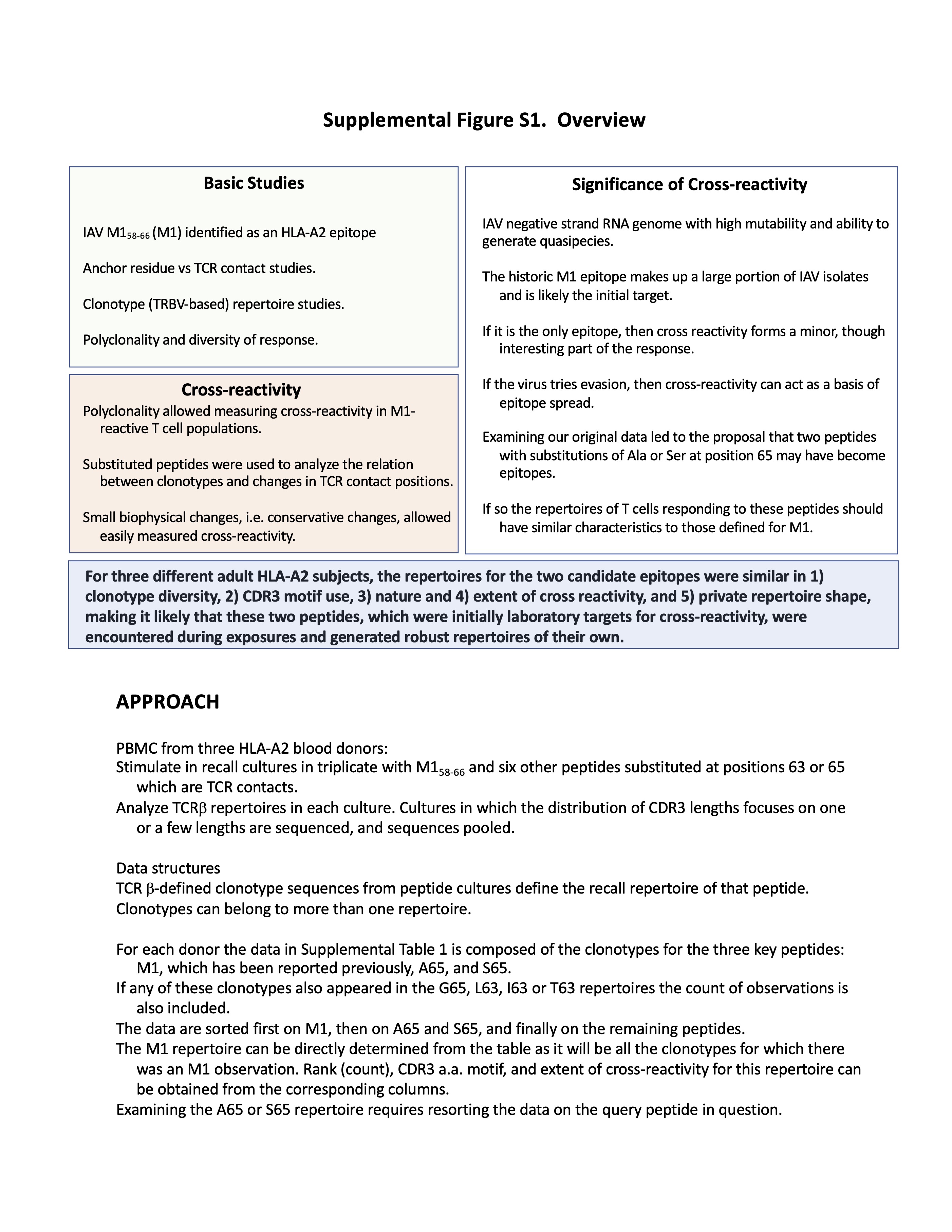

Supplement: Supplementary Figure 1 — Overview of paper. [file Image_1.jpg]

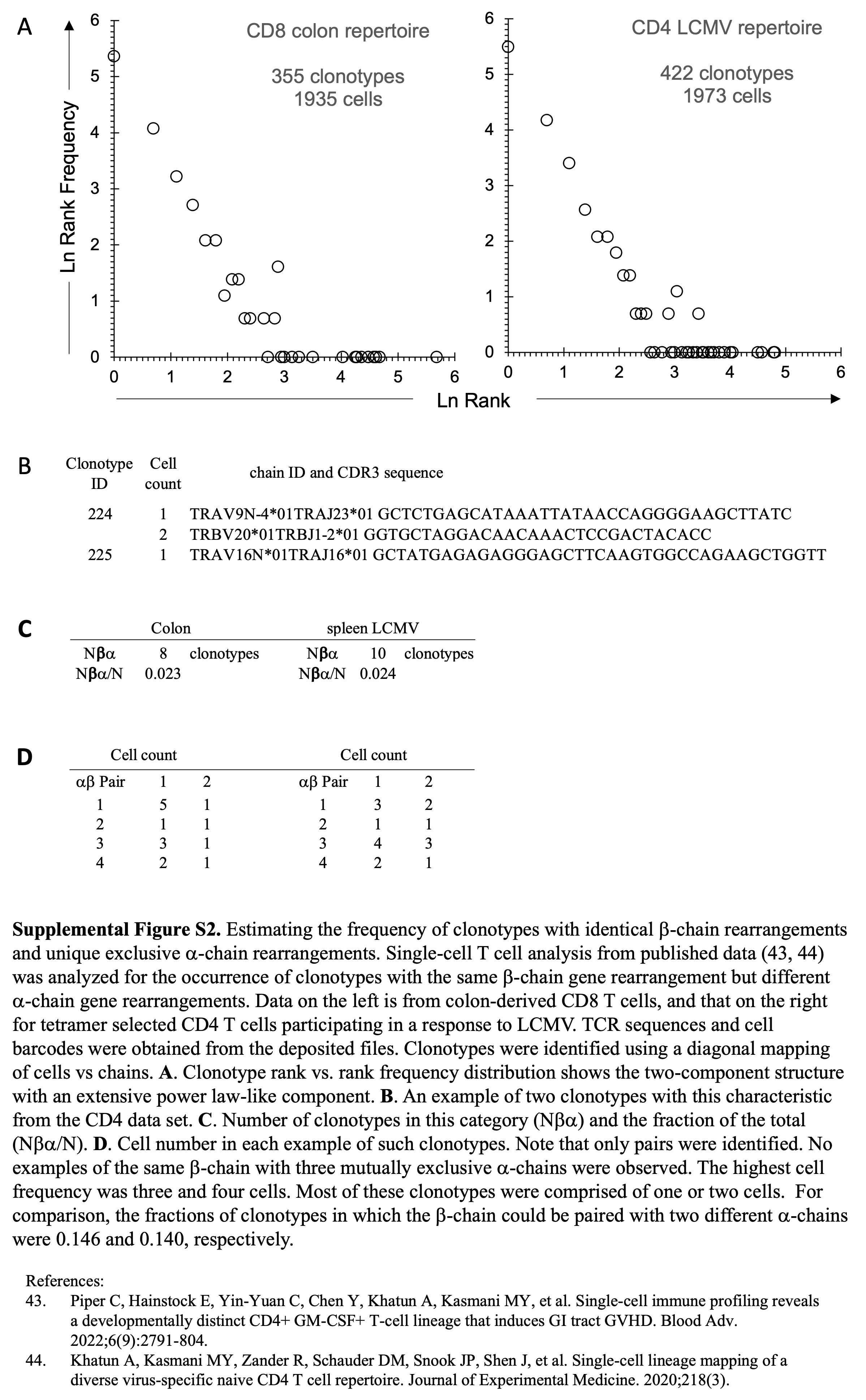

Supplement: Supplementary Figure 2 — Estimating the frequency of clonotypes with identical β-chain rearrangements and unique exclusive α-chain rearrangements. Single-cell T cell analysis from published data (43, 44) was analyzed for the occurrence of clonotypes with the same β-chain gene rearrangement but different α-chain gene rearrangements. Data on the left is from colon-derived CD8 T cells, and that on the right for tetramer selected CD4 T cells participating in a response to LCMV. TCR sequences and cell barcodes were obtained from the deposited files. Clonotypes were identified using a diagonal mapping of cells vs chains. (A) Clonotype rank vs. rank frequency distribution shows the two-component structure with an extensive power law-like component. (B) An example of two clonotypes with this characteristic from the colon CD4 data set. (C) Number of clonotypes in this category (Nβα) and the fraction of the total (Nβα/N). (D). Cell number in each example of such clonotypes. Note that only pairs were identified. No examples of the same β-chain with three mutually exclusive α-chains were observed. The highest cell frequency was three and four cells. Most of these clonotypes were comprised of one or two cells. For comparison, the fractions of clonotypes in which the β-chain could be paired with two different α-chains were 0.146 and 0.140, respectively. [file Image_2.jpg]
